# Transcatheter Intervention For Severe Aortic Coarctation in a Patient With Uninterrupted Left-Sided Inferior Vena Cava Presenting With Recurrent Abdominal Pain

**DOI:** 10.7759/cureus.8204

**Published:** 2020-05-19

**Authors:** Wail Alkashkari, Faisal Al-Husayni, Mohammed Althobaiti, Attafah Omeish, Saad A Alqahtani

**Affiliations:** 1 Cardiology, King Faisal Cardiac Center, King Abdulaziz Medical City, Ministry of National Guard Health Affairs, Jeddah, SAU; 2 Cardiology, King Abdullah International Medical Research Center, King Saud Bin Abdulaziz University for Health Sciences, Jeddah, SAU; 3 Internal Medicine, National Guard Hospital, King Abdulaziz Medical City, Jeddah, SAU; 4 Radiology, King Abdulaziz Medical City, Ministry of National Guard Health Affairs, Jeddah, SAU; 5 Radiology, King Abdullah International Medical Research Center, King Saud Bin Abdulaziz University for Health Sciences, Jeddah, SAU

**Keywords:** coarctation, congenital heart disease, inferior vena cava, stenting, balloon

## Abstract

We describe a case of a 17-year-old male patient who was admitted to the hospital for an evaluation of his recurrent postprandial abdominal pain and fatigue on exertion. He was discovered to have severe post-ductal aortic coarctation (CoA) and uninterrupted left-sided inferior vena cava (IVC) draining into the right atrium crossing anterior to the abdominal aorta. There were no signs of IVC compression. Patient symptoms improved dramatically after CoA stenting on follow up. The presence of uninterrupted left-sided IVC in this particular case created a diagnostic dilemma, and it was of great importance to know such anomaly before the procedure. This association of uninterrupted left-sided IVC with CoA is unusual, and to our knowledge, our case is the first to report such congenital association.

## Introduction

Aortic coarctation (CoA) is the sixth most common congenital heart disease (CHD), accounting for 4%-8% of all CHD and occurs in 4 out of 1,000 live births with a male predominance. CoA can occur as an isolated lesion but is often associated with other cardiovascular lesions, such as a bicuspid aortic valve (BAV) in 50%-75% of the cases, aortic arch hypoplasia, subaortic stenosis, mitral valve abnormalities, ventricular and atrial septal defects, and patent ductus arteriosus [1,2]. On the other hand, congenital variations or anomalies of venae cavae are not uncommon, with a reported prevalence of up to 8.7% [3]. Common superior vena cava (SVC) anomalies are left-sided and duplicated SVC. Inferior vena cava (IVC) anomalies can be classified as pre-renal (interrupted IVC), renal (retro-aortic renal vein and circum-aortic venous callor), and post-renal (duplicated IVC, left-sided IVC, and retro caval ureter) [4]. Although these are usually without significant clinical implications, awareness of these anomalies is necessary to avoid diagnostic pitfalls and is an essential pre-operatively or pre-transcatheter intervention [4]. Few studies suggested a correlation between venae cavae anomalies and CoA, such as persistent left-sided SVC and CoA [5]. There is a reported case of interrupted left-sided IVC with hemiazygous continuation to persistent left-sided SVC in a patient with CoA [6]. In our case, the left-sided IVC was not interrupted; it was draining into the right atrium and was associated with CoA.

## Case presentation

**Figure 1:**
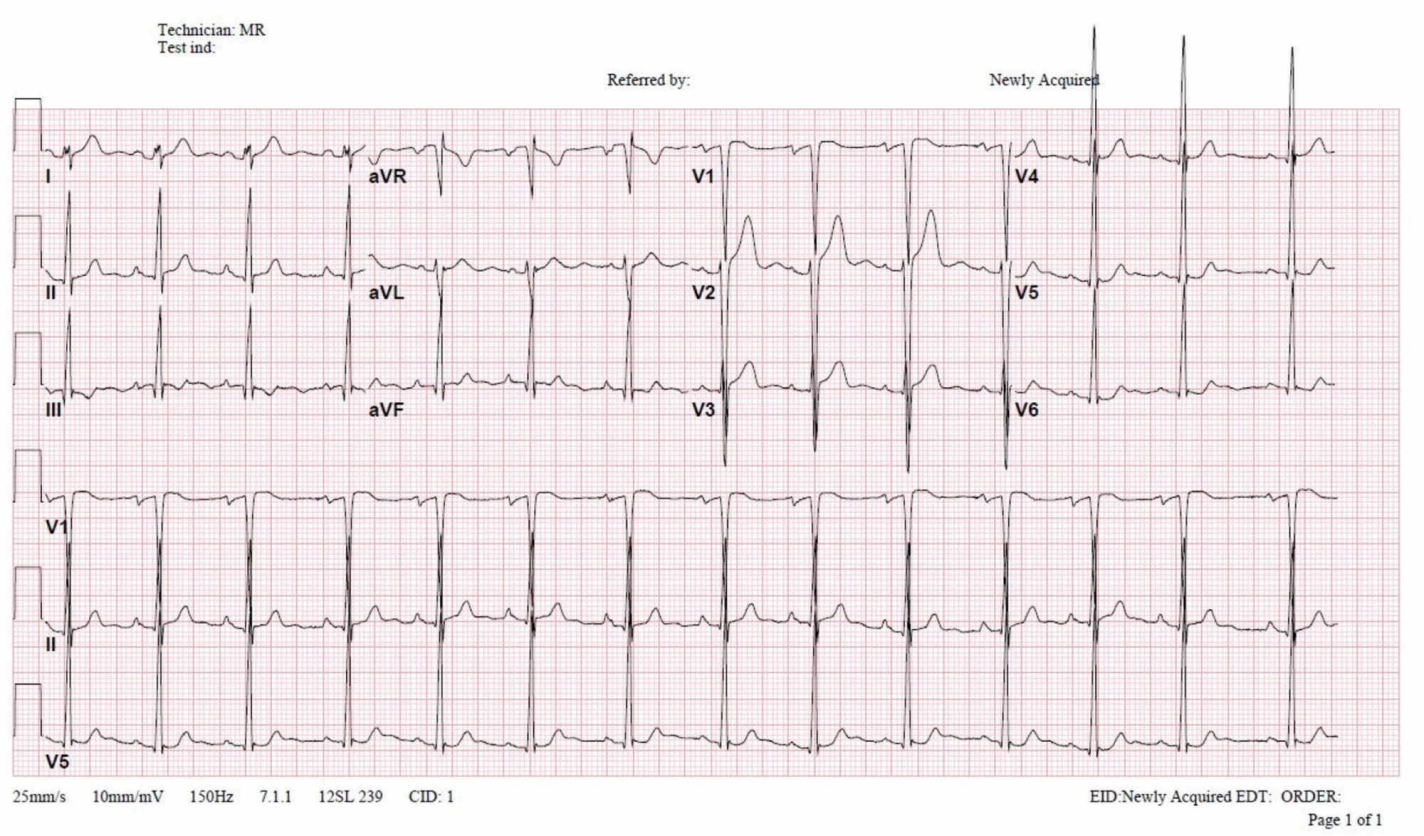
Electrocardiogram showing sinus rhythm and left ventricular hypertrophy.

**Figure 2:**
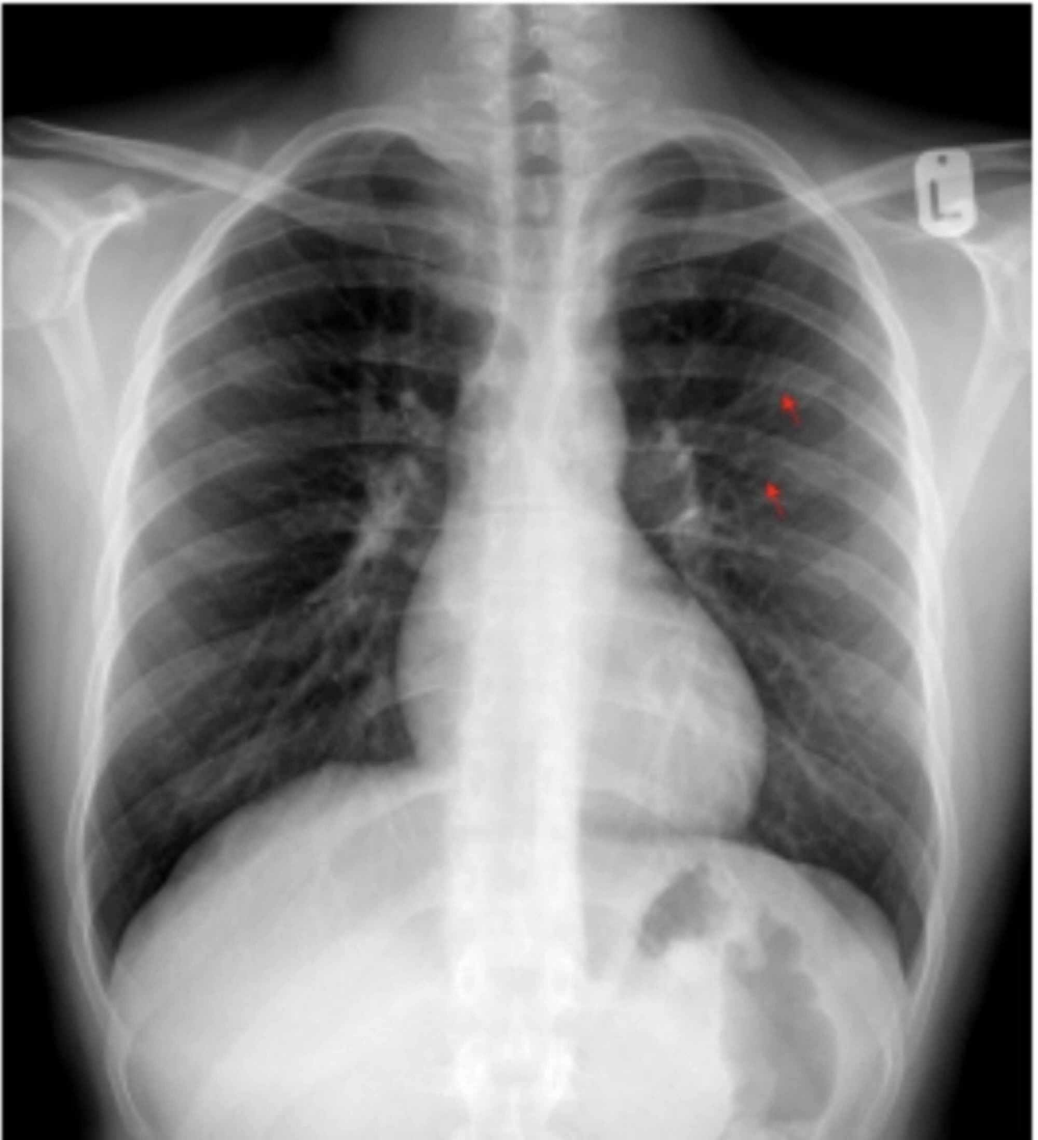
Chest X-ray showing classic rib-notching (red arrow).

**Figure 3:**
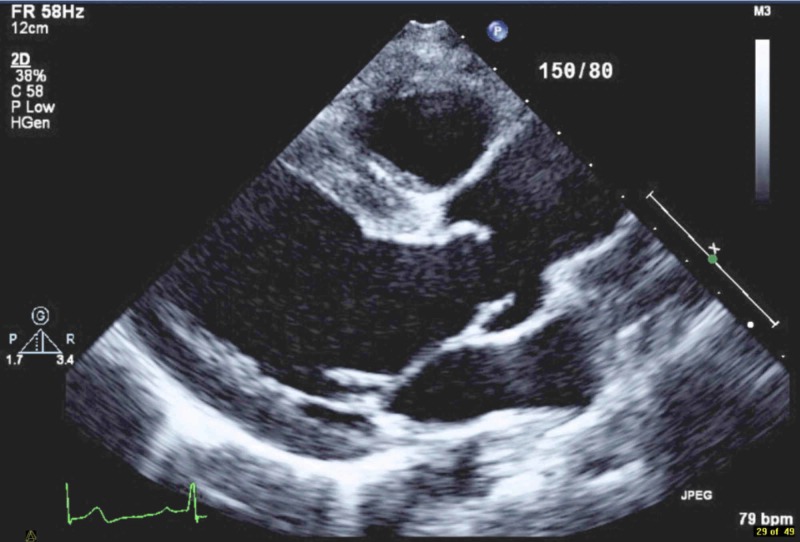
Parasternal long axis view showing bicuspid aortic valve and dilatation of ascending aorta.

**Figure 4:**
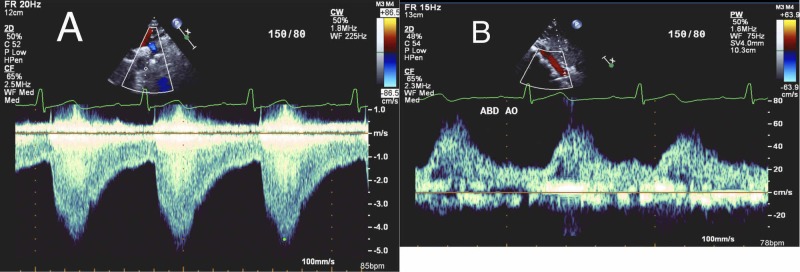
(A) Echocardiography showing post-ductal aortic coarctation with peak systolic gradient of 80 mmHg. (B) Doppler of the abdominal aorta showing continues diastolic flow.

**Figure 5:**
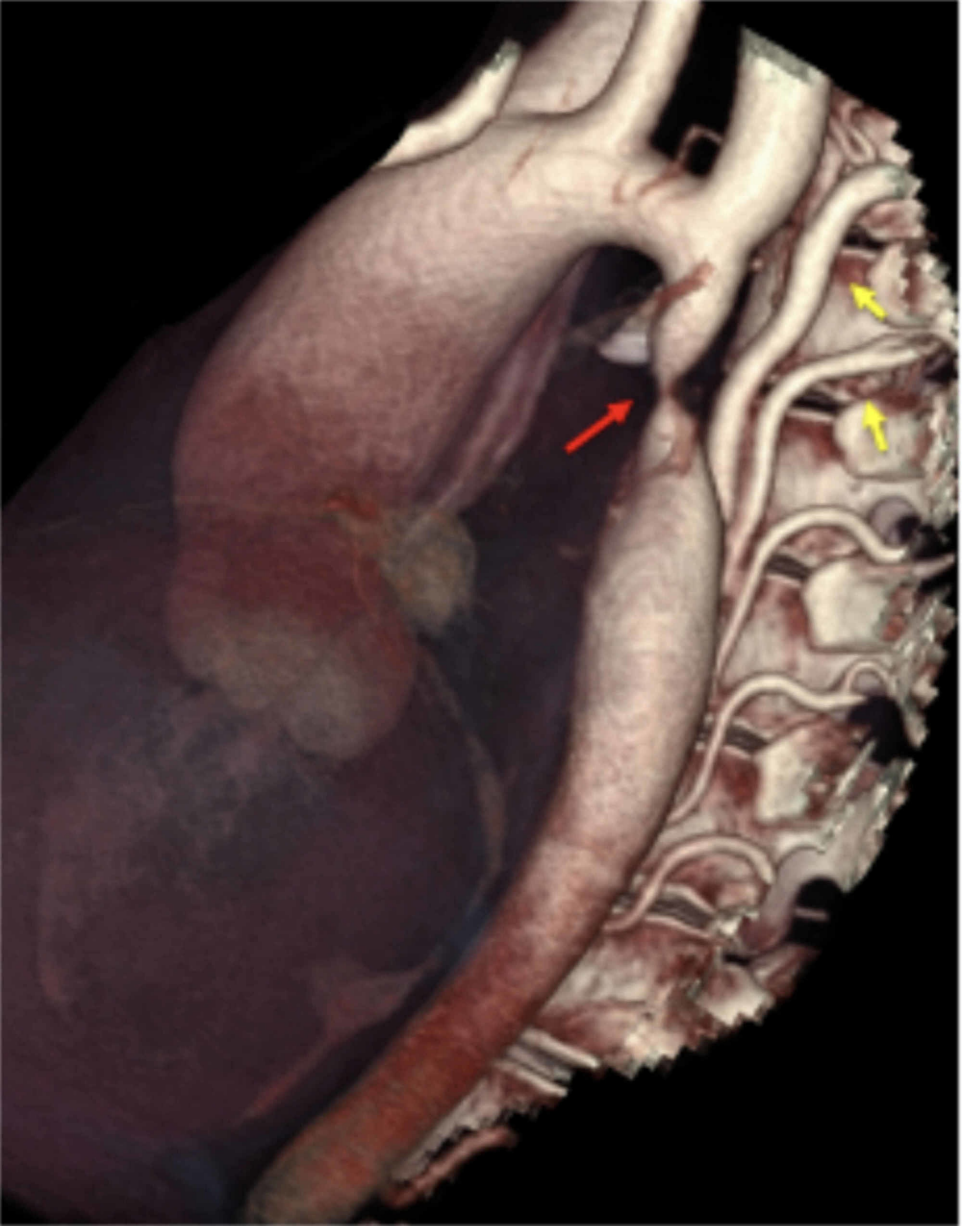
Three-dimensional volume rendering computed tomography of the aorta showing severe aortic coarctation (red arrow) and collaterals (yellow arrows).

**Figure 6:**
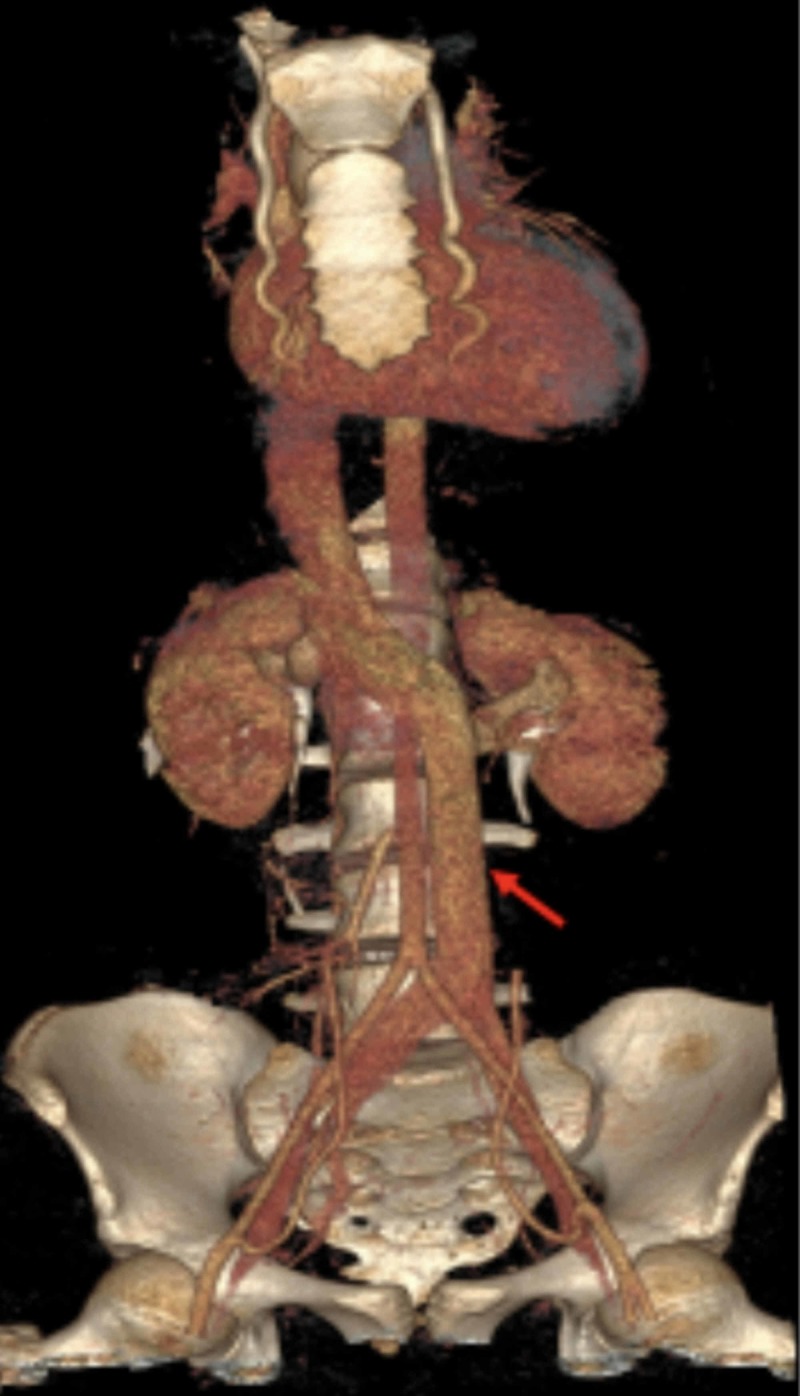
Three-dimensional volume rendering computed tomography showing the course of the left-sided inferior vena cava (red arrow).

A 17-year-old male with no previous medical illness was admitted to the hospital with abdominal pain. The patient had a long time history of generalized intermittent abdominal pain that increased postprandial without nausea or vomiting. The patient also had a history of fatigue on exertion. Upon checking vital signs, the patient was found to be hypertensive. There was a discrepancy between the upper (155-160/70-75 mmHg) and lower limbs (100-105/65-70 mmHg) readings. Cardiovascular examination showed a systolic ejection murmur best appreciated at the tip of the left scapula, and bracheo-femoral delay. The abdominal examination was unremarkable. His complete blood count, renal profile, liver profile, troponin, and brain natriuretic peptide were normal. Stool analysis and culture were normal. Electrocardiography revealed sinus rhythm and left ventricular (LV) hypertrophy (Figure 1). The chest X-ray revealed the classic rib notching sign (Figure 2). An echocardiogram revealed a BAV with mild to moderate aortic regurgitation and dilatation of ascending aorta (Figure 3). A post-ductal CoA was observed with a systolic gradient of 80 mmHg (Figure 4). His LV function was normal with mild eccentric hypertrophy. Cardiac CT was done and confirmed severe post-ductal CoA with extensive collaterals (Figure 5). Another incidental finding was a left-sided IVC, which courses anterior to the abdominal aorta between the aorta itself and superior mesenteric artery crossing the midline to the right before joining the right renal vein and the intra-hepatic IVC segment (Figure 6). The patient was discussed in a heart team meeting, and it was decided to proceed with transcatheter intervention with stenting.

**Figure 7:**
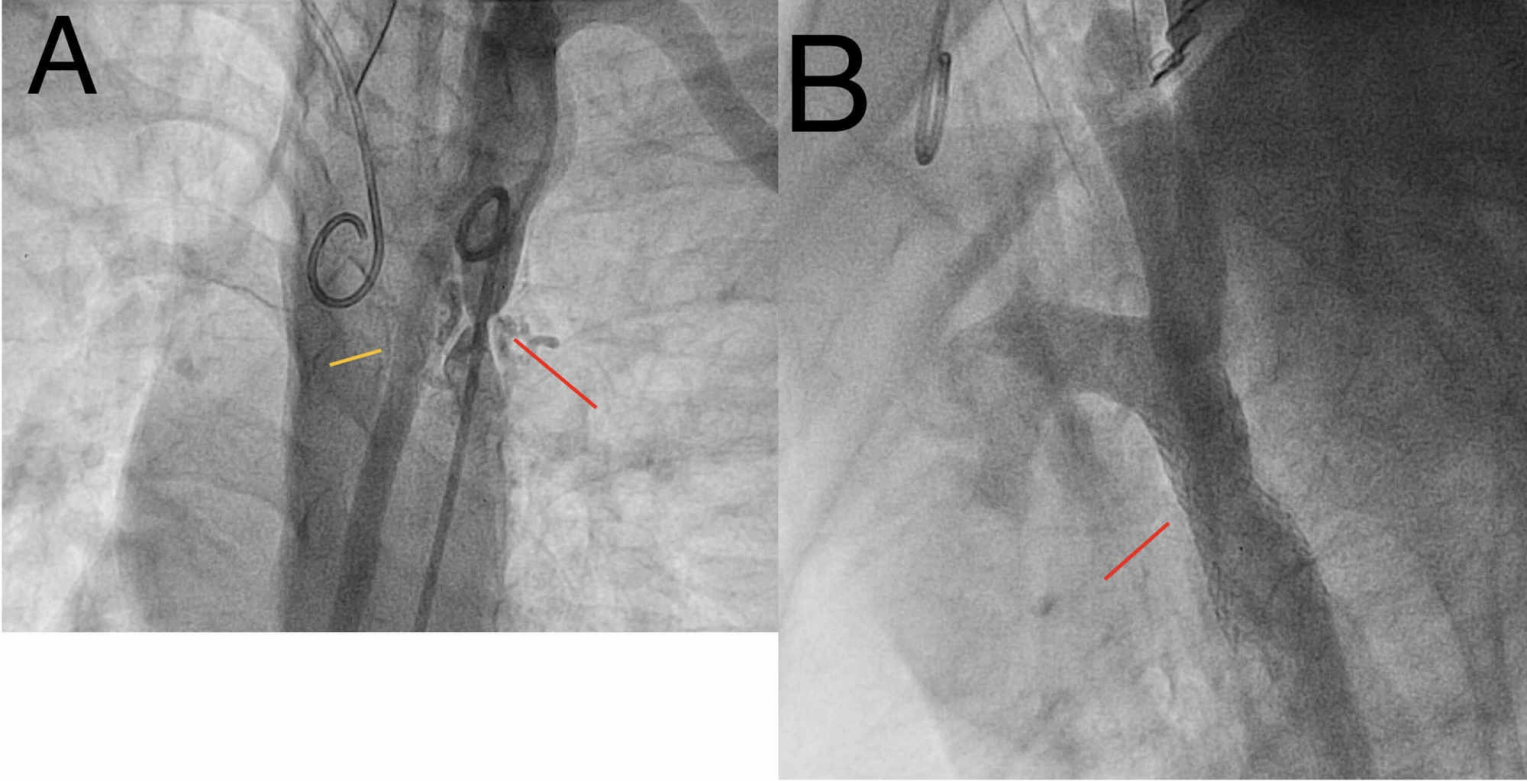
(A) Angiogram showing severe aortic coarctation (red arrow) and extensive collaterals (yellow arrow). (B) Angiogram showing post-coarctation stenting.

The procedure was done under general anesthesia through the right femoral access. The pre-stenting peak gradient was 60 mmHg, which improved to 4 mmHg using 14 x 39 mm BeGraft aortic stent (Bentley InnoMed, Hechingen, Germany), which is a balloon-expandable covered stent. This was followed by post-stenting dilation with short non-compliant Z-Med balloon (B. Braun Interventional Systems, Bethlehem, PA) (Figure 7). No temporary pacing was used for stenting. No periprocedural complications were encountered. The patient stayed in the hospital for two days, then was eventually discharged home on losartan 100 mg daily. Six weeks later, the patient was called for follow up. His symptoms resolved utterly, and blood pressure was well controlled.

## Discussion

CHD can occur as an isolated entity or in combinations. Some of the combinations are common and can predict the presence of each other such as BAV and CoA [1]. The company of left-sided IVC with CoA is unusual and very rare. Contrary to CoA, left-sided IVC is usually asymptomatic, and the diagnosis is incidental. Many cases have described left-sided IVC during imaging for malignancy staging, pulmonary embolism testing, and investigating vague symptoms such as abdominal pain and diarrhea [3,7]. Interestingly, symptoms are usually explained by other etiologies other than left-sided IVC despite having few cases of nutcracker syndrome due to entrapment of the left-sided IVC in the aorto-mesenteric edge [8]. In our case, the patient had abdominal pain, which is mostly related to CoA, but the possibility of venous congestion and nutcracker syndrome was present; however, it was ruled out by CT scan and supported by symptoms resolution after CoA repair. Obtaining femoral venous access in the transcatheter repair of CoA is not uncommon, and is usually done to perform a hemodynamic study and for the possibility of rapid right ventricular pacing during stent deployment [1]. Without knowing such an anomaly, the patient would be at risk of IVC perforation during catheter or pacemaker wire maneuvering through the IVC. The interventionist must be aware and cautious of such anomalies to manage the procedure delinquently.

## Conclusions

Congenital anomalies such as left-sided IVC are quite rare and usually do not require any intervention. However, the presence of such an abnormality can cause a diagnostic dilemma and create a challenge when managing other conditions that co-exist. We reported a case of a young man with uninterrupted left-sided IVC and CoA who underwent successful trans-catheter stent implantation. The association of uninterrupted left-sided IVC with CoA is unusual, and to our knowledge, our case is the first to report such a congenital association.
